# AMPA Receptor Antagonist NBQX Decreased Seizures by Normalization of Perineuronal Nets

**DOI:** 10.1371/journal.pone.0166672

**Published:** 2016-11-23

**Authors:** Wen Chen, Yan-Shuang Li, Jing Gao, Xiao-Ying Lin, Xiao-Hong Li

**Affiliations:** 1 Department of Neurology, Jinan Central Hospital, Affiliated to Shandong University, Jinan, Shandong Province, China; 2 Department of Neurology, The Affiliated Hospital Of Qingdao University, Qingdao, Shandong Province, China; Radboud Universiteit, NETHERLANDS

## Abstract

Epilepsy is a serious brain disorder with diverse seizure types and epileptic syndromes. AMPA receptor antagonist 2,3-dihydroxy-6-nitro-7-sulfamoyl-benzoquinoxaline-2,3-dione (NBQX) attenuates spontaneous recurrent seizures in rats. However, the anti-epileptic effect of NBQX in chronic epilepsy model is poorly understood. Perineuronal nets (PNNs), specialized extracellular matrix structures, surround parvalbumin-positive inhibitory interneurons, and play a critical role in neuronal cell development and synaptic plasticity. Here, we focused on the potential involvement of PNNs in the treatment of epilepsy by NBQX. Rats were intraperitoneally (i.p.) injected with pentylenetetrazole (PTZ, 50 mg/kg) for 28 consecutive days to establish chronic epilepsy models. Subsequently, NBQX (20 mg/kg, i.p.) was injected for 3 days for the observation of behavioral measurements of epilepsy. The *Wisteria floribundi agglutinin* (*WFA*)-labeled PNNs were measured by immunohistochemical staining to evaluate the PNNs. The levels of three components of PNNs such as tenascin-R, aggrecan and neurocan were assayed by Western blot assay. The results showed that there are reduction of PNNs and decrease of tenascin-R, aggrecan and neurocan in the medial prefrontal cortex (mPFC) in the rats injected with PTZ. However, NBQX treatment normalized PNNs, tenascin-R, aggrecan and neurocan levels. NBQX was sufficient to decrease seizures through increasing the latency to seizures, decrease the duration of seizure onset, and reduce the scores for the severity of seizures. Furthermore, the degradation of mPFC PNNs by chondroitinase ABC (ChABC) exacerbated seizures in PTZ-treated rats. Finally, the anti-epileptic effect of NBQX was reversed by pretreatment with ChABC into mPFC. These findings revealed that PNNs degradation in mPFC is involved in the pathophysiology of epilepsy and enhancement of PNNs may be effective for the treatment of epilepsy.

## Introduction

Epilepsy is a serious life-shortening neurological disorder, which affects approximately 1% of general population [1] and leads to an increase of disability and mortality in the world [2]. Epilepsy is a diverse phenotype including more than 15 different seizure types and more than 30 epilepsy syndromes [3]. The core symptom of epilepsy is recurring, unprovoked seizures induced by the abnormal synchronous activity of cerebral neuronal networks. The abnormal hypersynchronous activity induced by impaired inhibition increased extracellular potassium and enhanced excitatory synaptic transmission that are involved in the pathological process in epilepsy [4].

Glutamate is a predominant neurotransmitter released from excitatory neurons related to the fast synaptic excitation [5]. The released glutamates diffuse across the synaptic cleft and generate fast excitatory synaptic potentials (EPSPs) through binding to ionotropic glutamate receptors. The cumulative EPSPs of individual neurons trigger action potentials, which are responsible for epileptic field potentials. Glutamate receptors of the AMPA (a-amino-3-hydroxy-5-methyl-4-isoxazolepropionic acid) subtype have been evidenced to play a key role in epileptogenesis [6]. AMPA receptors regulate the fast synaptic excitation in brain regions that are related to epilepsy [7]. Specifically, AMPA receptor antagonists markedly reduce epileptiform and inhibit spread of epileptic discharges in both animal seizure models and human epilepsy [8,9]. In addition, growing evidence validate the critical role of AMPA receptors in epileptic seizures, and suggest that AMPA receptors may be a potential target for epilepsy therapy. Rodent models of epilepsy is used and it showed that perampanel, a potent, selective, orally active non-competitive AMPA receptor antagonist, exhibited a strong antiseizure activity in the maximal electroshock seizure test, and the 6 Hz seizure test [10,11]. Moreover, clinical data showed that perampanel with daily dose is effective in patients with refractory partial-onset seizures [12–14]. NBQX (2,3-dihydroxy-6-nitro-7-sulfamoyl-benzoquinoxaline-2,3-dione) is a competitive AMPA receptor antagonist and has a property that suppressed focal electrographic seizures in epileptic mice [9]. Recently, it was described that NBQX blocks the development of spontaneous recurrent seizures when treatment after neonatal seizures [15]. Consistently, noncompetitive AMPA antagonist perampanel improved the performance of partial seizures, suggesting a remarkable antiepileptogenic effect [16].

Perineuronal nets (PNNs) are condensed extracellular matrix (ECM) structures, which surround parvalbumin-positive inhibitory interneurons and play a critical role in neuronal cell development, activity and growth [17–19]. Chondroitin sulfate proteoglycans (CSPGs) are crucial components of PNNs [20], which have been suggested to be a critical factor regulating synaptic plasticity [21]. Aberrant PNN signaling was found to induce the dysfunctions of central nervous system such as epilepsy, stroke, Alzheimer’s disease, schizophrenia and addiction [22,23]. The plant lectins *Wisteria floribunda* agglutinin (*WFA*) can be used to visualize PNNs by binding to N-acetylgalactosamine residue [24]. Therefore, we use *WFA*-labeled neurons to evaluate the numbers of PNNs in the current investigation. The three major ECM components of PNNs are tenascin-R, aggrecan and neurocan, which are important contributors to formation and stabilization of PNNs functions [25].

Pentylenetetrazole (PTZ), a g-aminobutyric acid antagonist, is widely used to establish experimental models simulate human epilepsy. PTZ administration increased the glutamatergic transmitter and induced generalized tonic–clonic seizures in animals with higher doses [26,27]. In the current study, we used PTZ at the dose of 50 mg/kg (i.p.) to induce chronic seizures to observe the anti-epileptic effect of NBQX and the possible involvement of PNNs.

## Materials and Methods

### Animals

Male Wistar rats that weighed 220–240 g upon arrival were obtained from the Shandong University Experimental Animal Center and were used for all experiments. Rats were individually housed under constant temperature (23±2°C) and humidity (50±5%) and maintained on a 12-hour light/dark cycle with free food and water available. Rats were monitored daily by an experimenter at 8:00–12:00 am each day for eating, drinking, and activity, which were used for the physical health assessment. There are no animals died before the experimental endpoint. All of the animal procedures were performed in accordance with the National Institutes of Health Guide for the Care and Use of Laboratory Animals. The protocol of current animal experiments was approved by the Ethics of Animal Experiments of Jinan Central Hospital (Permit Number: SA-2014-008). All surgery was performed under sodium pentobarbital anesthesia, and all efforts were made to minimize suffering in animals. All of the behavioral tests and drug administrations were carried out in the dark phase.

### Drugs

AMPAR antagonist 2,3-dihydroxy-6-nitro-7-sulfamoyl-benzoquinoxaline-2,3-dione (NBQX), pentylenetetrazole (PTZ) and chondroitinase ABC (ChABC) were supplied from Sigma-Aldrich Chemical Company (Sigma, St Louis, Missouri, USA). NBQX was freshly dissolved in saline as sodium salt. ChABC were dissolved in 0.1 m PBS (vehicle) for microinjection into the medial prefrontal cortex and prepared in stock solutions of 0.02 U/μl.

### Seizures induction and behavioral analysis

**Fig 1:**
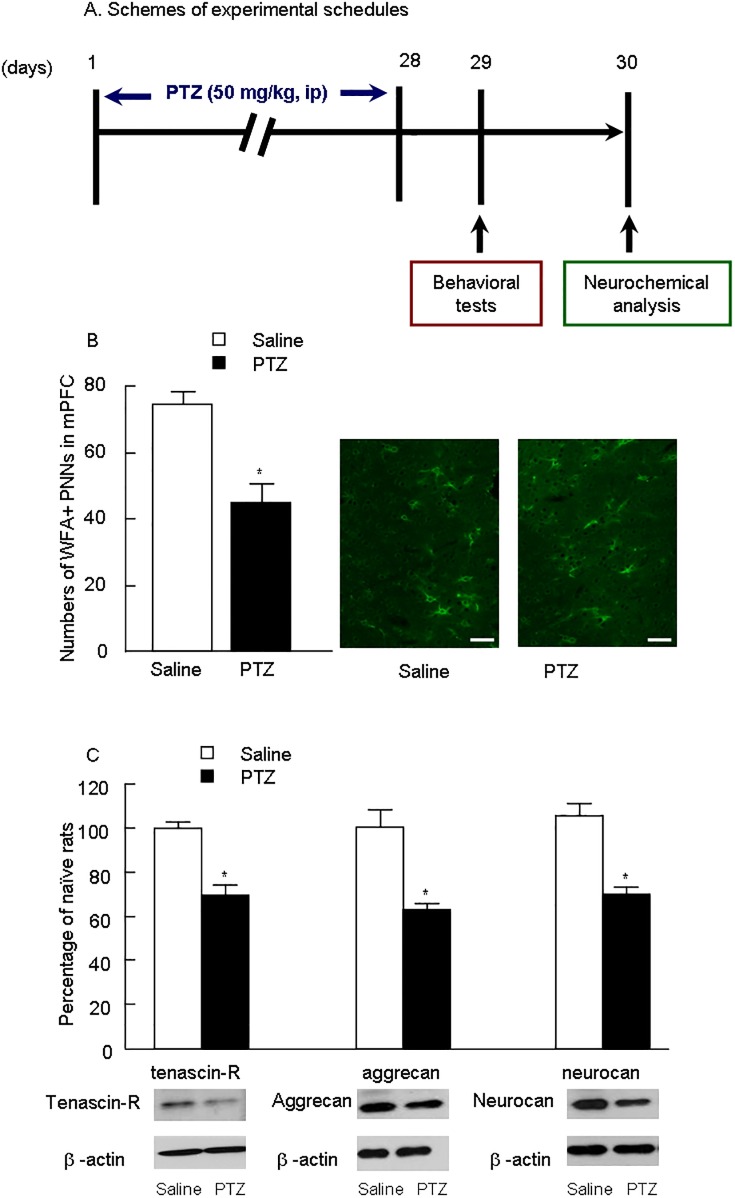
Chronic PTZ treatment reduced PNNs (*WFA*), tenascin-R, aggrecan and Neurocan in the medial prefrontal cortex. (A) Schemes of experimental schedules. (B) Numbers of *WFA*+ PNNs in mPFC of control and PTZ treatment, scale bar is 50 μm, n = 6 per group. Representative *WFA*+ images of immunofluorescence staining are shown on the right. The data are expressed as mean ± SEM. (C) The levels of tenascin-R, aggrecan and Neurocan in mPFC are shown. Representative Western blot images are shown on the right. The data are expressed as a percentage of the values obtained for the rats treated with saline. *p < 0.01, different from corresponding saline groups (*n =* 6).

Rats were deprived of food but not water 12h before the experiments to prevent aspiration of food. Rats in PTZ group were treated with a single 50 mg/kg intraperitoneal (i.p.) administration of PTZ for 28 days. While rats were injected with saline (i.p.) in control group instead. Behavioral tests and neurochemical analysis were performed on day 29 and 30, respectively (Fig 1A).

**Fig 2:**
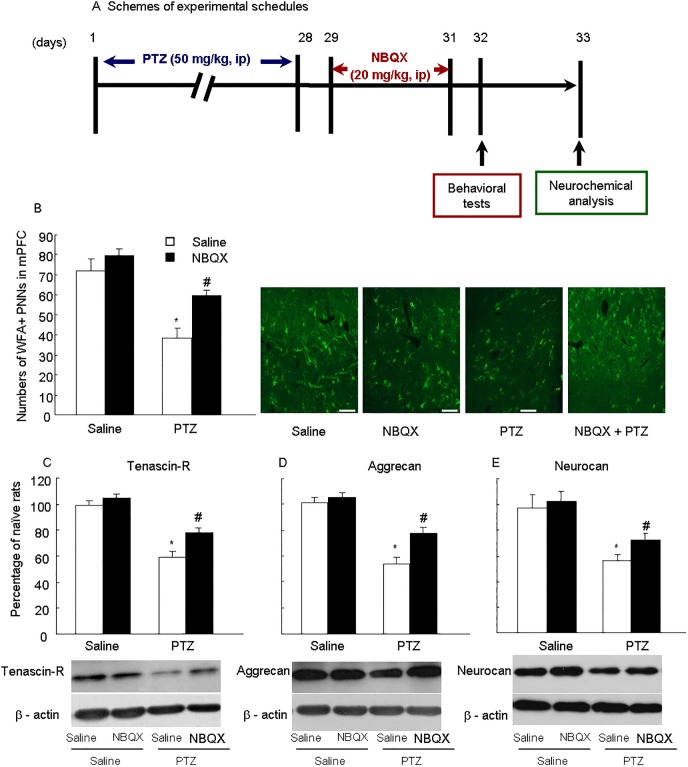
NBQX increased PNNs (*WFA*), tenascin-R, aggrecan and Neurocan in the medial prefrontal cortex in PTZ-treated rats. (A) Schemes of experimental schedules. (B) Numbers of *WFA*+ PNNs in mPFC of control and PTZ treatment, scale bar 50 μm, n = 6 per group. Representative *WFA*+ images of immunofluorescence staining are shown on the right. The data are expressed as mean ± SEM. The levels of (C) tenascin-R, (D) aggrecan and (E) Neurocan in mPFC are shown. Representative Western blot images are shown on the right. The data are expressed as a percentage of the values obtained for the rats treated with saline. *p < 0.01, different from corresponding saline groups, # *p* < 0.01, compared with PTZ group (*n =* 6).

To observe anti-epileptic effects of NBQX in PTZ induced epilepsy, we divided rats into four groups: rats in saline + saline group were treated with saline only; rats in PTZ + saline group were treated with 50 mg/kg of PTZ (i.p.) and saline for 28 days; rats in saline + NBQX group were treated with saline for 28 days, and 20 mg/kg of NBQX (i.p.) for next 3 days; rats in PTZ + NBQX group were treated with 50 mg/kg of PTZ (i.p.) for 28 days and were treated with 20 mg/kg of NBQX (i.p.) for next 3 days. Behavioral tests and neurochemical analysis were performed on the following 2 days (Fig 2A). The doses for PTZ and NBQX were selected regarding to previous studies [15,28].

**Fig 3:**
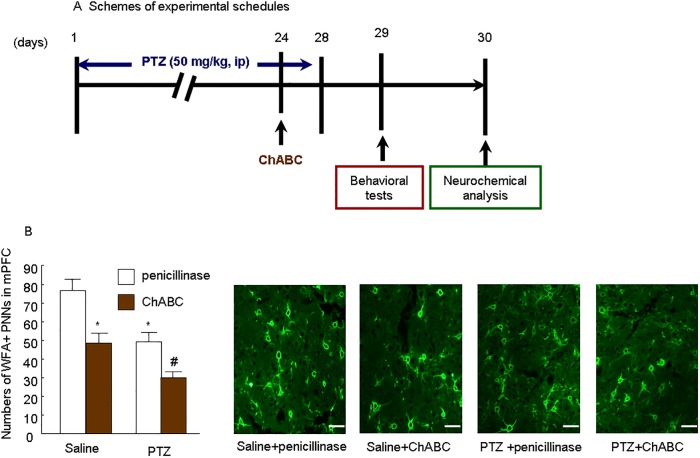
PNNs (*WFA*) were removed by ChABC microinjection into mPFC. (A) Schemes of experimental schedules. (B) Numbers of *WFA*+ PNNs in mPFC of control and PTZ treatment, scale bar 50 μm, n = 6 per group. Representative *WFA*+ images of immunofluorescence staining are shown on the right. The data are expressed as mean ± SEM. *p < 0.01, compared with saline group; # *p* < 0.01, compared with PTZ group.

To clarify the effects of degradation of PNNs caused by ChABC in mPFC on the seizures, we used four groups of rats: saline + penicillinase group was treated with saline plus penicillinase into mPFC on d24; saline + ChABC group was treated with saline and microinjection of ChABC (0.01 U/μg/side/0.5 μl) into mPFC on d24; PTZ + penicillinase group was treated with PTZ (50 mg/kg) for 28 days and microinjected with penicillinase on d24; PTZ + ChABC group was treated with PTZ for 28 days and microinjected with ChABC on d24. Behavioral tests and neurochemical analysis were performed on the following 2 days (Fig 3A).

To determine whether PNNs degradation by ChABC can reverse the anti-epileptic effect of NBQX, we injected rats with PTZ for 28 days and separated them into four groups: rats in vehicle group were treated with vehicle without NBQX, and were microinjected with penicillinase into mPFC on d 24; rats in vehicle + ChABC group were treated with vehicle plus microinjection of ChABC into mPFC on d24; rats in NBQX + penicillinase group were treated with penicillinase on d24 and were treated with NBQX injection on d 29 to d31; rats in NBQX + ChABC group were treated with microinjection of ChABC into mPFC on d24 plus NBQX (20 mg/kg, i.p.) on d 29 to d31. Behavioral tests were performed on day 32.

The epileptic seizure activity induced by PTZ was evaluated by latency to seizures (s), duration of the minor seizure onset (s), duration of the major seizure onset (s), and scores for the severity of seizures in 1h after PTZ injection [28]. The minor seizure onset was termed as isolated myoclonic jerks and clonic seizures accompanied by facial and front extremity muscle clonus. While the major seizure following the minimal seizure are characterized by head, neck, and tail extension with the loss of the tonic flexor reflex and tonic flexion–extension following the protracted clonus [29]. The scores were used to measure the severity of seizures after PTZ administration according to the following level: 0: no changes in behavior; 1: isolated myolonic jerks; 2: only atypical minimal seizures; 3: minimal seizures; 4: major seizures without a tonic phase; and 5:completed tonic–clonic seizures [30]. The performance of each rat was recorded by a video camera during the entire experimental procedures. The observers for the measurement of seizure scores were blind to the treatment of each group.

### Immunofluorescence and image analysis

After the behavioral measurement, rats were deeply anesthetized with sodium pentobarbital (100 mg/kg, i.p.) and were intracardially perfused with 200–250 ml of 0.1M phosphate-buffered saline, pH 7.4, followed by 200–250 ml of 4% paraformaldehyde phosphate buffer, pH 7.4. The brains were then postfixed at 4°C for 24 h and dehydrated in 30% sucrose for at least 4 days. Serial coronal 30 μm brain sections that contained the medial prefrontal cortex were cut on a Leica freezing microtome and stored in a cryoprotectant solution at -20°C. The sections were incubated overnight at 4°C in a solution of biotin-conjugated lectin *wisteria floribunda* (*WFA*, Sigma Aldrich, L1516). All of the sections were then washed 3 times in PBS and then incubated in FITC-conjugated streptavidin (Sigma-Aldrich, S3762) in 25°C for 3 h. Four or five sections from each brain region of each rat were selected. Fluorescence microscope with an image-analysis program was used for measuring the number of *WFA*-positive PNNs. The average number of PNNs on either side of target brain region was taken as the positive immunoreactive cell number for each rat as previously reported [31,32].

### Tissue sample preparation

Rats were killed 30 min after the last administration of PTZ. Their brains were extracted and removed. Subsequently, bilateral tissue punches of the mPFC (16 gauge) were obtained from approximately 1 mm thick coronal sections cut in a Reichert-Jung 2800 Frigocut E cryostat at -20°C. The rostral faces of the coronal sections were approximately 3.8 mm from bregma. Tissue punches were homogenized (10–15 s × 3, 5 s interval) with an electrical disperser (Wiggenhauser, Sdn Bhd) after being lysed with RIPA lysis buffer with protease-inhibitor (Beyotime Biotechnology, Beijing, China) for 30 min. Afterward, the homogenate was subjected to 10,000 × *g* centrifugation at 4°C for 20 min. All of the above procedures were performed under low temperature (0–4°C). The protein concentrations of all samples were determined using the BCA assay kit (Beyotime Biotechnology). The protein concentration was normalized by diluting the samples with RIPA lysis buffer.

### Western blot assays

Samples were subjected to sodium dodecyl sulfate-polyacrylamide gel electrophoresis (8% acrylamide/0.27% *N*,*N’*-methylenebisacryalamide resolving gel) for approximately 30 min at 80 V in stacking gel and approximately 1 h at 120 V in resolving gel. Proteins were transferred electrophoretically to Immobilon-P transfer membranes (Millipore, Bedford, MA, USA) at 0.25 A for 3 h. Membranes were washed with TBST (Tris-buffered saline plus 0.05% Tween-20, pH 7.4) before dipping in blocking buffer (5% skimmed dry milk in TBST) overnight at 4°C. Membranes were then incubated for 1 h at room temperature with anti-tenascin-R (1:400; Santa Cruz, sc-9875), anti-aggrecan (1:400, Santa Cruz, sc-25674), anti-neurocan (1:1000, Sigma Aldrich, N0913), and anti-β-actin antibody (1:2000, A5316; Sigma, St. Louis, MO, USA) in TBST plus 5% bovine serum albumin. After the membrane was shaken in 4 × 6 min washes in TBST buffer, the blots were incubated for 45 min at room temperature with horseradish peroxidase-conjugated secondary antibody (goat anti-rabbit or mouse IgG; Santa Cruz Biotechnology and Vector Labs, respectively) diluted 1:5000 in blocking buffer. The blots were then shaken in 4 × 6 min washes in TBST. Afterward, the blots were incubated with a layer of Super Signal enhanced chemiluminescence substrate mixture (Pierce Biotechnology, Rockford, IL, USA) for 1 min at room temperature. Finally, the blots were exposed against X-ray film (Eastman Kodak Company). Band intensities were quantified using Quantity One software (version 4.0.3) from Bio-Rad Corporation (Hercules, CA, USA).

### Intracerebral cannula implantation and intracranial injections

Rats were anesthetized with sodium pentobarbital (60 mg/kg, i.p.), and guide cannulae (23-gauge, Plastics One, Roanoke, VA, USA) were implanted bilaterally 1 mm above the medial prefrontal cortex with the following stereotaxic coordinates: anterior/posterior (A/P), -3.2 mm; medial/lateral (M/L), ± 2.5 mm; dorsal/ventral (D/V), -3.3 mm [33–35]. The rats were allowed to recover for at least 7 d before intracranial injections. Vehicle or ChABC (0.01 U/μg/side/0.5 μl) were intracranial microinjected using 10 μl Hamilton syringes (Hamilton, Reno, NV, USA) connected via polyethylene-50 tubing to 30-gauge injectors (Plastics One) into the mPFC. The dose of ChABC was used as previously evidenced [31]. A total volume of 0.5 μl was infused into each side over 1 min, and the injection syringe was left in place for an additional 1 min to allow for diffusion. At the end of the behavioral tests, the rats were anesthetized with sodium pentobarbital (100 mg/kg, i.p.) and transcardially perfused. Cannula placements were assessed using Nissl staining with a thickness of 30 μm under a light microscope to determine the infusion site. Subjects with misplaced cannulae were excluded from the statistical analysis.

### Data analysis

The data are expressed as mean ± SEM and were analyzed using one- or two-way analysis of variance (ANOVA) followed by Tukey’s *post hoc* test (for details, see Results section). In the two-way ANOVA tests, two factors are involved: 1. PTZ and saline groups, 2. NBQX and saline, or ChABC and vehicle treatment. Values of P < 0.05 were considered statistically significant.

## Results

### The behavioral patterns of epileptic seizures induced by PTZ

Chronic PTZ treatment induced significant changes on latency to seizures, duration of the minor seizure onset, duration of the major seizure onset, and scores for the severity of seizures in rats. As shown in Table 1, rats in saline group produced a longer latency (600 s) to seizures than those in PTZ group (102.9 ± 6.5 s, *p* < 0.001). Additionally, PTZ treatment also induced an increase in the duration of both minor seizure onset (65.1 ± 4.4 s *vs* 0 s, *p* < 0.001) and major seizure onset (117.8±12.9 s *vs* 0 s, *p* < 0.001) compared with saline group, respectively. Similarly, the scores for the severity of seizures were increased by PTZ injection (4.6 ± 0.26). These data suggest that rats chronically treated with PTZ exhibited a marked phenotype of epilepsy model.

**Table 1:** PTZ induced significant changes on latency to seizures (s), duration of the minor seizure onset (s), duration of the major seizure onset (s), and scores for the severity of seizures in rats.

Group	Saline	PTZ	*p* value
N	8	8	
Latency to seizure (s)	600	102.9±6.5	<0.001
Duration of the minor seizure onset (s)	0	65.1±4.4	<0.001
Duration of the major seizure onset (s)	0	117.8±12.9	<0.001
Scores for the severity of seizures	0	4.6±0.26	<0.001

### Chronic PTZ treatment reduced WFA, tenascin-R, aggrecan and neurocan in mPFC

After behavioral analysis, rats were killed and their brains were collected for the following assay for PNNs (Fig 1A). The data from immunofluorescence staining showed that PTZ significantly decreased the number of *WFA*+ PNNs in mPFC compared to saline-treated rats (Fig 1B, *p* < 0.001). Furthermore, Western blot assay revealed that the levels of three components of PNNs, tenascin-R, aggrecan and neurocan (Fig 1C, *p* < 0.001) were also reduced in PTZ group compared with saline group, suggesting that the PNNs was impaired by chronic PTZ administration.

### AMPA receptor antagonist NBQX decreased seizures induced by PTZ

Next, we aimed to identify the effect of AMPA receptor antagonist NBQX on the onset of epileptic seizures induced by PTZ. Data from behavioral tests in Table 2 showed that the decreased latency to seizure induced by PTZ was increased by NBQX treatment (*p* < 0.01). Moreover, the increases in duration of the minor seizure onset (*p* < 0.01), duration of the major seizure onset (*p* < 0.01), and the scores for the severity of seizures (*p* < 0.01) in PTZ group were completely reduced by NBQX injection. Two-way ANOVA with the between-subjects factors PTZ (0 and 50 mg/kg) and NBQX (0 and 20 mg/kg) revealed significant effects of PTZ (*F*1,28 = 94.5, *p* < 0.001) and NBQX (*F*1,28 = 16.5, *p* < 0.001) on the latency to seizure and a PTZ × NBQX interaction (*F*1,28 = 16.5, *p* < 0.001). In the duration of the minor seizure onset measurement, two-way ANOVA revealed significant effects of PTZ (*F*1,28 = 163.1, *p* < 0.001) and NBQX (*F*1,28 = 13.9, *p* < 0.01), and a PTZ × NBQX interaction (*F*1,28 = 13.9, *p* < 0.001). In consistent, two-way ANOVA revealed significant effects of PTZ (*F*1,28 = 234.9, *p* < 0.001) and NBQX (*F*1,28 = 18.9, *p* < 0.001), and a PTZ × NBQX interaction (*F*1,28 = 18.9, *p* < 0.001) on the duration of the major seizure onset. Two-way ANOVA also showed significant effects of PTZ (*F*1,28 = 108.8, *p* < 0.001) and NBQX (*F*1,28 = 21.5, *p* < 0.001), and a PTZ × NBQX interaction (*F*1,28 = 21.5, *p* < 0.001) on the scores for the severity of seizures. These results indicate that treatment with AMPA receptor antagonist NBQX effectively reversed the behavioral abnormality of epileptic seizures of chronic PTZ administration in rats.

**Table 2:** NBQX decreased seizures induced by PTZ on latency to seizure (s), duration of the minor seizure onset (s), duration of the major seizure onset (s), and scores for the severity of seizures in rats.

Group	Saline	Saline+NBQX	PTZ	PTZ+NBQX
N	8	8	8	8
Latency to seizure (s)	600	600	108.3±8.2*	178.6±15.2#
Duration of the minor seizure onset (s)	0	0	67.6±6.0*	37.0±5.6#
Duration of the major seizure onset (s)	0	0	122.0±10.3*	68.1±6.8#
Scores for the severity of seizures	0	0	4.5±0.27*	2.9±0.23#

* p<0.01, compared with saline group;

^#^ p<0.01, compared with PTZ group.

### NBQX normalized WFA, tenascin-R, aggrecan and neurocan in mPFC

We further assessed the effects of NBQX on the PNNs in the medial prefrontal cortex (Fig 2A). Rats exposed to NBQX produced a significant increase in the positive staining of *WFA*-labeled neurons, which was reduced by chronic PTZ treatment (Fig 2B, *p* < 0.01). Two-way ANOVA analysis showed that significant effects of PTZ (*F*1,20 = 33.1, *p* < 0.001) and NBQX (*F*1,20 = 9.6, *p* < 0.01), and a PTZ × NBQX interaction (*F*1,20 = 6.2, *p* < 0.05) on the *WFA* positive stainings. We also measured the three important components of PNNs and found that the reductions of tenascin-R (*p* < 0.01), aggrecan (*p* < 0.01) and neurocan (*p* < 0.01) in mPFC of PTZ group were increased by NBQX treatment (Fig 2C–2E). The results suggest that the anti-epileptic action of NBQX might be through prevention of the reduction of PNNs by increasing the active components tenascin-R, aggrecan and neurocan.

### The degradation of PNNs caused by ChABC in mPFC exacerbated seizures

To further examine the regulatory role of PNNs in the development and treatment of epileptic seizures, we used ChABC to degrade PNNs in the mPFC and subsequently measured the behavioral patterns in PTZ-treated rats. ChABC microinjection into mPFC decreased PNNs (*WFA*), and co-treatment with PTZ strengthened this decrease (Fig 3A and 3B). The behavioral results showed that intra-mPFC injection of ChABC alone did induce the onset of seizures with decreased latency to seizure (*p* < 0.01), increased duration of the minor (*p* < 0.01) and major seizure onset (*p* < 0.01), and increased scores for the severity of seizures (*p* < 0.01) in rats (Table 3). Moreover, when ChABC and PTZ were treated simultaneously to one rat, the onset of seizures was more serious than those two drugs were used alone (Table 3). Two-way ANOVA with the between-subjects factors PTZ (0 and 50 mg/kg) and ChABC (0 and 0.02 U) revealed significant effects of PTZ (*F*1,28 = 78.1, *p* < 0.001) and ChABC (*F*1,28 = 23.1, *p* < 0.001) on the latency to seizure and a PTZ × ChABC interaction (*F*1,28 = 6.5, *p* < 0.05). In the duration of the minor seizure onset measurement, two-way ANOVA revealed significant effects of PTZ (*F*1,28 = 31.8, *p* < 0.001) and ChABC (*F*1,28 = 18.2, *p* < 0.001), and a PTZ × ChABC interaction (*F*1,28 = 7.4, *p* < 0.01). In consistent, two-way ANOVA revealed significant effects of PTZ (*F*1,28 = 52.9, *p* < 0.001) and ChABC (*F*1,28 = 23.7, *p* < 0.001), and a PTZ × ChABC interaction (*F*1,28 = 4.9, *p* < 0.05) on the duration of the major seizure onset. Two-way ANOVA also showed significant effects of PTZ (*F*1,28 = 66.7, *p* < 0.001) and ChABC (*F*1,28 = 51.8, *p* < 0.001), and a PTZ × ChABC interaction (*F*1,28 = 5.6, *p* < 0.05) on the scores for the severity of seizures. These results revealed that degradation of PNNs in the mPFC exacerbated the epileptic seizure induced by PTZ, suggesting that the dysfunction of PNNs might be involved in the induction of epilepsy.

**Table 3:** ChABC exacerbated the epileptic seizure induced by PTZ.

Group	Saline+penicillinase	Saline+ChABC	PTZ+penicillinase	PTZ+ChABC
N	8	8	8	8
Latency to seizure (s)	600	506.9±25.4*	110.5±6.1*	70.1±9.5#
Duration of the minor seizure onset (s)	0	8.5±1.5*	63.9±5.4*	84.0±3.6#
Duration of the major seizure onset (s)	0	18.4±2.8*	123.6±10.1*	167.6±7.4#
Scores for the severity of seizures	0	1.6±0.26*	4.1±0.22*	5#

* p<0.01, compared with saline group;

^#^ p<0.01, compared with PTZ group.

### ChABC reversed the anti-epileptic effects of NBQX in PTZ-induced seizures

We next examined the potential of degradation of PNNs on the anti-epileptic effects of NBQX in PTZ-induced seizures. ChABC, NBQX and their vehicles were injected into four groups of rats after receiving PTZ treatment. Two-way ANOVA with the between-subjects factors NBQX (0 and 20 mg/kg) and ChABC (0 and 0.02 U) revealed significant effects of NBQX (*F*1,28 = 30.3, *p* < 0.001) and ChABC (*F*1,28 = 14.2, *p* < 0.01) on the latency to seizure and a NBQX × ChABC interaction (*F*1,28 = 6.7, *p* < 0.05). In the duration of the minor seizure onset measurement, two-way ANOVA revealed significant effects of NBQX (*F*1,28 = 47.9, *p* < 0.001) and ChABC (*F*1,28 = 29.5, *p* < 0.001), and a NBQX × ChABC interaction (*F*1,28 = 7.1, *p* < 0.01). In consistent, two-way ANOVA revealed significant effects of NBQX (*F*1,28 = 16.6, *p* < 0.001) and ChABC (*F*1,28 = 8.3, *p* < 0.001), and a NBQX × ChABC interaction (*F*1,28 = 5.6, *p* < 0.05) on the duration of the major seizure onset. Two-way ANOVA also showed significant effects of NBQX (*F*1,28 = 20.8, *p* < 0.001) and ChABC (*F*1,28 = 10.6, *p* < 0.01), and a NBQX × ChABC interaction (*F*1,28 = 5.1, *p* < 0.05) on the scores for the severity of seizures. NBQX significantly increased the latency to seizures, and decreased the duration of the minor seizure onset, the duration of the major seizure onset, and the scores for the severity of seizures (Table 4). However, ChABC blocked the anti-epileptic effects of NBQX in PTZ-induced seizures, suggesting that normalization of PNNs in the medial prefrontal cortex might underlie the therapeutic action of NBQX.

**Table 4:** ChABC reversed the anti-epileptic effects of NBQX in PTZ-induced seizures.

Group	Vehicle+penicillinase	Vehicle+ChABC	NBQX+penicillinase	NBQX+ChABC
N	8	8	8	8
Latency to seizure (s)	109.3±6.3	98.1±4.3	184.6±14.8*	125.4±8.3#
Duration of the minor seizure onset (s)	62.6±4.8	79.1±1.4*	37.6±3.4*	57.6±3.0#
Duration of the major seizure onset (s)	121.0±8.7	133.9±8.2	78.9±7.7*	111.5±6.9#
Scores for the severity of seizures	4.3±0.16	4.6±0.18	3.1±0.23*	4.0±0.19#

* p<0.01, compared with saline group;

^#^ p<0.01, compared with PTZ group.

## Discussion

The present study showed that chronic PTZ treatment induced significant changes of seizures in rats by reducing PNNs (*WFA*) numbers, and the components tenascin-R, aggrecan and neurocan in mPFC. However, AMPA receptor antagonist NBQX decreased the onset of seizures through increasing PNNS in mPFC. Moreover, degradation of PNNs by ChABC in mPFC exacerbated seizures and reversed the anti-epileptic effects of NBQX in PTZ-treated rats. Thus, the present findings demonstrate the regulatory role of PNNs in mPFC on the development and treatment of epilepsy. The deficits of PNNs measured by reduced *WFA* numbers and decreased tenascin-R, aggrecan and neurocan protein levels might be related to the pathophysiology of epilepsy induced by PTZ.

The critical roles of the AMPA receptors in epileptic seizures and anti-epileptic treatment have been previously demonstrated [36,37]. Several selective AMPA receptor antagonists were demonstrated to have broad spectrum anticonvulsant activity in sound-induced seizures in rats and in amygdala-kindled rats [38,39]. Similarly, a decrease of phosphorylated AMPA glutamate receptor subunit (GluA1) was found in the dorsal hippocampus of rats after chronic pilocarpine treatment [40]. In addition, AMPA receptor antagonists were widely found to be safe and effective in patients with partial-onset seizures [41], raising a possibility that AMPA receptor can be a novel target for epilepsy therapy. NBQX, a potent and selective AMPA receptor antagonist, produced effective anticonvulsant protection against sound-induced seizures in mice [42]. Furthermore, systematic administration with NBQX at the dose of 10–40 mg/kg exhibited strong antiepileptogenic and anticonvulsant actions on kindling in rats [43]. In the present study, we used PTZ to induce chronic epileptic seizures in rats and found that NBQX effectively reversed the behavioral abnormality of epileptic seizures. However, NBQX (40 mg/kg, ip) effectively increased latency to seizures, decreased duration of the minor and major seizure onset, and reduced scores for the severity of seizures in rats. AMPA receptors, located at excitatory synapses, are involved in the initiation and synchronization of epileptic discharges by excitatory neurons [44]. Therefore, selective blockade of AMPA receptor-related glutamatergic neurotransmission may inhibit neuronal excitability and produce antiepileptic properties in chronic epilepsy.

PNNs inhibit structural rearrangements at synapses, and consequently contribute to the maintenance of neuronal networks [19,45]. PNN-expressing perisomatic interneurons also express AMPA receptors with specific subunits. The excitatory postsynaptic currents (EPSC) are extremely fast and are mediated by AMPA receptors, thus enabling interneurons to detect and shape synchronous activity of principal cells [46]. It has been evidenced that the charge of the synaptic membrane shapes the AMPAR-mediated current by influencing the clearance of the negatively charged glutamate from the synapse [47]. The structural and molecular organization of PNNs is heterogeneous and depends on the neuronal cell types consisting of tenascin-R, aggrecan and neurocan that enwraps the perikaryon of several neurons [48,49]. PNNs dysfunction has been implicated in several neuropsychiatric disorders, including epilepsy [50,51]. Brain-specific CSPG includs aggrecan and neurocan in the developing and mature rat brain and has been demonstrated to control axonal extension or regeneration via inhibition of neuronal cell migration and neurite extension from surviving neurons [52–54]. Tenascin-R, a member of the tenascin gene family of extracellular matrix proteins, is synthesized by oligodendrocytes with high expression levels during the period of active myelination [55]. It has been demonstrated that tenascin-R is associated with an episode of pilocarpine-induced status epilepticus through modulation of mossy fiber sprouting and astrogliosis [54,56]. Furthermore, systemic administration of kainic acid (KA) caused changes in neurocan leading to neuronal degeneration in the limbic structures [51]. We found that PTZ destroyed PNNs in mPFC with epileptic behaviors, while NBQX increased PNNs (*WFA*), tenascin-R, aggrecan and neurocan and exerted anti-epileptic effects. Our results confirmed previous evidence that prolonged changes in tenascin-R and neurocan in the remodeling of neuronal networks are related to establishment or enhancement of epileptogenesis. This data revealed a relationship between PNNs and epilepsy, and suggested that modulation of PNNs in critical brain region will provide novel therapeutic approaches for epilepsy.

A recent investigation suggested that chondroitin sulfate-degrading enzyme ChABC enhanced the lateral mobility of the AMPA receptor, and consequently promote short-term synaptic plasticity [57]. ChABC may degrade various PNNs components, and consequently destroy the PNNs structure. In the current study, pretreatment with ChABC blocked the anti-epileptic effects of NBQX in PTZ-induced seizures, suggesting that normalization of PNNs in mPFC might underlie the therapeutic action of AMPA receptor antagonist NBQX. Further study is needed to evaluate the effect of upregulation of PNNs in the mPFC on the epilepsy induced by PTZ and on the treatment benefits of NBQX. The finding that the fast movements of AMPA receptors are involved in the modulation of synaptic transmission suggests that AMPAR mobility regulates the availability of naive receptors for synapses. Previous studies showed that removal of the PNNs leads to an increase of AMPAR exchange between extrasynaptic and synaptic sites, and may modulate synaptic properties [57]. Our results revealed that chronic epilepsy induced a reduction of components of PNNs, tenascin-R, aggrecan and neurocan in mPFC, while AMPA receptor antagonist NBQX increased the levels of these proteins, suggesting that PNNs might be essential for the functionality of synaptic transmission. Thus, future studies targeting at PNNs can shed light on development of novel antiepileptic drugs with good efficacy and acceptable tolerability for therapy in epilepsy.

## Conclusion

In summary, the present data showed that AMPA receptor antagonist NBQX decreased the onset of epileptic seizures induced by PTZ through regulation of PNNs in the medial prefrontal cortex. Degradation of PNNs caused by ChABC in mPFC not only exacerbated seizures but also reversed the anti-epileptic effect of NBQX in PTZ-treated rats. These findings therefore revealed that PNNs in the medial prefrontal cortex is related to the anti-epileptic effect of NBQX and enhancement of PNNs may be effective for the treatment of epilepsy.
